# Christmas at the London, 1912

**Published:** 1913-01-04

**Authors:** 


### CHRISTMAS AT THE LONDON, 1912.

### "Mr." George and the Dragon.

Christmas Day at the London Hospital began, as it has begun for many years, at four o'clock in the morning with a long procession of sisters and nurses passing slowly through the wards and corridors singing the old-time Christmas carols. Some of them helped the carols with violin music, and most carried lanterns. The effect in the huge building is rather beautiful. Then follows routine work—breakfast and "getting up" for such as are able to, in order that all may be ready at nine o'clock for the advent of Father Christmas and his Comus Rout of clowns, nondescripts, and convalescent children dressed as fairies. Every patient in the hospital is provided with a present—a good, substantial, useful present, which has been specially packed and addressed by the ward sister and nurses over-night and placed in a great pile outside each ward, in order that Father Christmas and his minions may replenish their baskets on entry.

Father Christmas is always represented by the Senior Resident Doctor, who always, whatever his natural formation, contrives to assume the characteristic bulk and geniality necessary to the part. He is generally accompanied by several of the other resident doctors in various disguises, such as clowns, etc., a prominent feature this year being one disguised as what was variously held to be either "Man Friday" or the palæolithic "Man from Sussex." Probably the most salient feature of the procession, however, was an enormous dragon, some forty feet long, evolved by the fertile brain of Mr. Arthur Rackham, the well-known artist, and other kindred spirits. This Saurian, with movable jaws and eyes and a flexible tail, glorious in hues of green, scarlet, and yellow, was most ingeniously constructed by the registrars of the hospital, and its motive power was furnished by four stout clerks and dressers. A gallant St. George, in plate and chain mail, in ordinary life a burly registrar, put Leviathan through its paces; it was a huge success. Father Christmas's round takes about three hours, and is the hardest "round" of the year. Meantime in the children's wards stockings and Christmas trees have been in full swing. At twelve o'clock came dinner in each ward, the carving solemnly ascribed to the respective house surgeon or house physician, dressed in traditional cook's attire, who did the honours with due solemnity. Smoking followed in the men's wards of the hospital, and at about two o'clock began the first of a series of performances that followed one another in close succession until eight o'clock, when "lights out" are sounded. The entertainments are provided by numerous professional and volunteer artists and are of all kinds, but naturally the most popular are the performances of the various troupes made up of the medical students and resident doctors of the hospital; these deal lightly with topical events, with dignities and dignitaries—for example, the Hospital Band, composed of the house surgeons and physicians, the receiving-room officers, etc., had two chief episodes, the first dealing with the new electric notice board, which signals the arrival of the "Knuts." Among the "Knuts" special mention may be made of the Chairman, the Hon. Sydney Holland, and those members of the staff whose special characteristics lend themselves to immortality.

The second episode dealt with a humorous operation—a gorgeous burlesque, which no one appreciated more than those who had been operated upon. A telephone conversation with Mr. Lloyd George about his Insurance Act was also a great hit.

The other troupes of local talent comprised the "Royalists," an artistic performance by some magnificent cavaliers; the "Bronchi," an excellent cowboy show; and the "Italians," who gave a humorous performance with an organ and monkey. After the last of the ward performances there were further performances and a dance for the hospital female staff in the huge out-patient department. We were unable to make use of the new cinematograph on this occasion, as the programme had been already arranged, and this show could not be fitted in. As usual, everyone enjoyed themselves to the full, and no one is a penny the worse.

